# Imaging surrogate of the ventricular arrhythmia substrate in a swine model of ventricular tachycardia: high resolution LGE vs. high resolution electro-anatomical mapping

**DOI:** 10.1186/1532-429X-17-S1-Q133

**Published:** 2015-02-03

**Authors:** Sébastien Roujol, Tamer A Basha, Cory M Tschabrunn, Kraig V Kissinger, Mark E Josephson, Warren J Manning, Elad Anter, Reza Nezafat

**Affiliations:** 1 Department of Medicine (Cardiovascular Division), BIDMC / Harvard Medical School, Boston, MA, USA; 2 Radiology, BIDMC / Harvard Medical School, Boston, MA, USA

## Background

Assessment of risk of sudden cardiac death (SCD) due to ventricular tachycardia and fibrillation (VT/VF) remains one of the most challenging areas in management of patients with chronic myocardial infarction. VT substrate consists of reentry circuits near or within a chronic scar. Invasive catheter mapping using electro-anatomical system (EAM) is the clinical standard for identification of reentry circuits. However, current EAM systems have low spatial resolution which may contribute to the high recurrence rate of VT after ablation (50% at 2 years). Better identification of reentry circuits can potentially improve SCD risk assessment and outcome of VT ablation procedures. Late gadolinium enhancement (LGE) CMR has the potential for non-invasive identification of reentry circuits, but LGE sequences suffer from low spatial resolution and partial voluming (1). In this study, we sought to utilize a high-resolution 3D LGE with 1 mm^3^ isotropic spatial resolution to image the VT surrogate in swine model of VT.

## Methods

A 180 min balloon occlusion of the mid left anterior artery was induced in 6 Yorkshire swine. Each animal underwent an in-vivo CMR exam at 52±13 days after infarction. An electrophysiology study was subsequently performed using a novel proprietary high-resolution 64 electrode basket catheter (Rhythmia Medical) with programmed stimulation to assess for VT inducibility and full LV mapping with ~7000 sampling points. Both in-vivo and ex-vivo CMR of the excised heart were performed using a 1.5 T Philips scanner. An injection of 0.2 mmol/kg of gadobenate dimeglumine was used during in-vivo CMR and again at 20 min before euthanasia. High resolution LGE was performed using a free breathing navigator-gated inversion recovery gradient echo (GRE) sequence (TR/TE/α=6.5/3.0ms/25˚, FOV=270×270×112 mm^3^, voxel size=1×1×1 mm^3^, compressed sensing factor=4) and was reconstructed using a B_1_-weighted reconstruction based on LOST (2). Ex-vivo CMR included a T_1_-weighted acquisition with GRE imaging (TR/TE/α=15/7.1ms/25˚, FOV=130×130×100 mm^3^, voxel size=0.5×0.5×0.5 mm^3^, 3 averages). In-vivo LGE data were analyzed offline. Endocardial and epicardial contours of the myocardium were manually delineated. The transmural intensity, averaged over the inner 1 mm layer of the myocardium, was normalized (where ‘0' corresponded to 3 standard deviations above the mean signal intensity of normal myocardium and ‘1' corresponds to 60% of the maximum signal intensity of the scar area) and projected on the endocardial shell.

## Results

**Figure 1:**
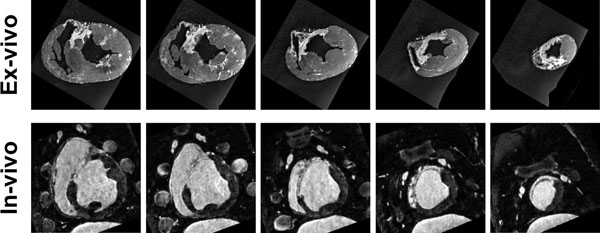
Example in-vivo (bottom row) high resolution 1 mm^3^ isotropic resolution LGE and ex-vivo T_1_-weighted acquisition (top row) with 0.5 mm^3^ isotropic resolution in a swine model of VT.

**Figure 2:**
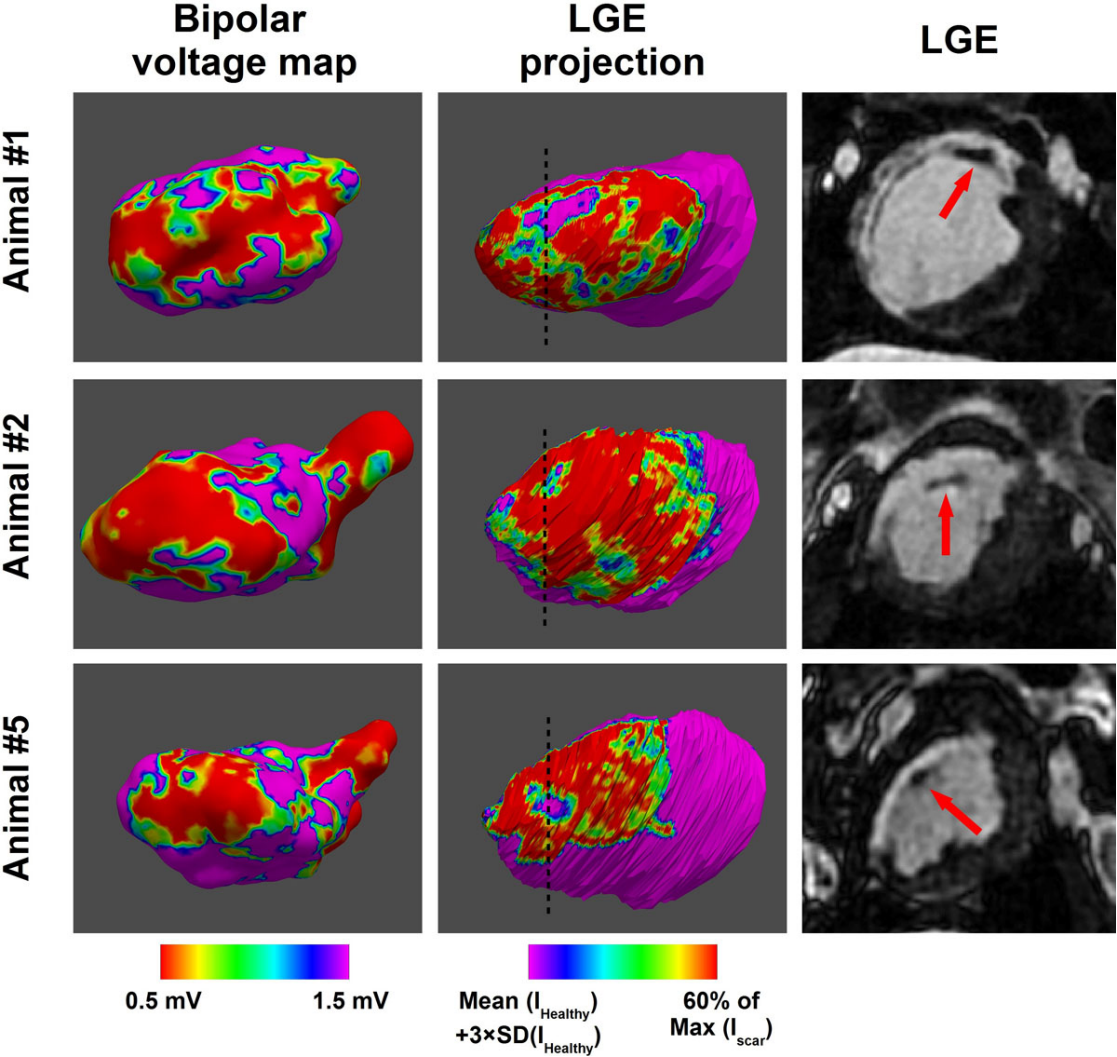
Bipolar voltage maps and in-vivo LGE obtained in three different swine. "LGE projection" (middle column) is the normalized average transmural intensity over the inner 1 millimeter layer of the myocardium. Viable myocardial located within the scar area can clearly be observed using high resolution LGE data. The overall scar area can be visualized in the septal area of the three swine in both bipolar voltage maps and LGE data. The core scar area (bipolar voltages < 0.5 mV and LGE normalized projection > 60% of Max(I_scar_)) is also matching between both datasets.

Sustained reentrant VT was induced in all animals. In-vivo high resolution LGE shows good visual correspondence with ex-vivo CMR data (Figure 1). The inner 1 millimeter layer of the myocardium in LGE data was found to visually match with high resolution EAM (Figure 2).

## Conclusions

High resolution LGE with 1 mm^3^ isotropic resolution enables imaging of a surrogate of the VT substrate as defined by high-resolution EAM.
